# Current Understanding of the Role of Cholesterol in the Life Cycle of Alphaviruses

**DOI:** 10.3390/v13010035

**Published:** 2020-12-29

**Authors:** Ivanildo P. Sousa, Carlos A. M. Carvalho, Andre M. O. Gomes

**Affiliations:** 1 Laboratório de Enterovírus, Instituto Oswaldo Cruz, Fundação Oswaldo Cruz, Rio de Janeiro 21040-900, Brazil; 2 Centro de Ciências Biológicas e da Saúde, Departamento de Patologia, Universidade do Estado do Pará, Belém 66095-662, Brazil; c_marques@live.com; 3 Seção de Arbovirologia e Febres Hemorrágicas, Instituto Evandro Chagas, Ananindeua 67030-000, Brazil; 4 Instituto Euro-Americano de Educação, Centro Universitário Metropolitano da Amazônia, Ciência e Tecnologia, Belém 66053-000, Brazil; 5 Centro de Ciências da Saúde, Instituto de Bioquímica Médica Leopoldo de Meis, Universidade Federal do Rio de Janeiro, Rio de Janeiro 21941-902, Brazil; amog@bioqmed.ufrj.br; 6 Centro Nacional de Biologia Estrutural e Bioimagem, Instituto Nacional de Ciência e Tecnologia de Biologia Estrutural e Bioimagem, Universidade Federal do Rio de Janeiro, Rio de Janeiro 21941-902, Brazil

**Keywords:** alphavirus, cholesterol, fusion, lateral organization, membrane

## Abstract

Enveloped viruses rely on different lipid classes present in cell membranes to accomplish several steps of their life cycle in the host. Particularly for alphaviruses, a medically important group of arboviruses, which are part of the Togaviridae family, cholesterol seems to be a critical lipid exploited during infection, although its relevance may vary depending on which stage of the virus life cycle is under consideration and whether infection takes place in vertebrate or invertebrate hosts. In this review, the role of cholesterol in both early and late events of alphavirus infection and how viral replication may affect cholesterol metabolism are summarized, taking into account studies on Old World and New World alphaviruses in different cell lines. Moreover, the importance of cholesterol for the structural stability of alphavirus particles is also discussed, shedding light on the role played by this lipid when they leave the host cell.

## 1. Introduction

Alphaviruses are arboviruses belonging to the *Togaviridae* family and are broadly distributed on all continents, where they are transmitted between vertebrate hosts mainly by mosquito vectors. Depending on where they occur, alphaviruses have been traditionally classified as belonging to either Old World or New World types [1]. In general, Old World alphaviruses, such as Ross river virus (RRV) and Chikungunya virus (CHIKV), are arthritogenic, while New World alphaviruses, such as Venezuelan equine encephalitis virus (VEEV) and Eastern/Western equine encephalitis viruses (EEEV/WEEV), are encephalitogenic [2]. The former group usually causes less severe disease and has lower mortality rates in humans relative to the latter group, and, with few exceptions, there are no vaccines or antivirals available to control these agents [1].

Alphaviruses usually have a wide spectrum of possible hosts, making the jump between unrelated species a common event in the maintenance of these viruses in the wild; most of the more than 30 known alphavirus species are transmitted alternately between mosquito vectors and vertebrate hosts including humans, nonhuman primates, equids, birds, amphibians, reptiles, rodents, and pigs [3].

Alphaviruses consist of enveloped particles about 70 nm in diameter containing an unsegmented, single-stranded RNA genome of 9.7–12 kb enclosed by a distinct icosahedral core and a transmembrane glycoprotein layer [3]. The virions are composed of three major structural proteins (C, E1, and E2) in a 1:1:1 stoichiometric ratio, producing a highly stable and symmetrical particle, where the C protein is directly associated with the RNA genome forming the nucleocapsid, while E1 and E2 proteins make up trimers of heterodimers on the viral envelope [4]. Depending on the alphavirus species, three additional structural proteins (E3, 6K, and TF) may also be present in the virion, but these are not required for the particle to be infectious [5].

Although the alphavirus genome also codes for four nonstructural proteins (nsP1, nsP2, nsP3, and nsP4), they are supposed to exist only during virus replication in the infected cell, regardless of the host species, playing a critical role at this stage [6]. However, a recent study based on mass spectrometry surprisingly detected that nsP2 is also incorporated within Sindbis virus (SINV) grown in multiple cells representing vertebrate (BHK-21, HEK293, and HepG2 cells) and mosquito (C7-10 cells) hosts, suggesting a role for this protein during packaging and/or entry of progeny virus. Furthermore, some host proteins—such as sorting nexin 5 (SNX5)—were found to be associated with highly purified SINV particles, although in lower abundance [7].

Particularly important for the structure and function of alphaviruses, the lipid composition of the viral envelope varies depending on whether replication took place in vertebrate or invertebrate host cells, especially with respect to cholesterol [8].

## 2. Understanding the Role of Cholesterol in Biological Membranes

Since its discovery and isolation in the middle of the 18th century, cholesterol has been extensively studied. With its intricate and complex biosynthesis system and metabolism, the molecule initially attracted the attention of biochemists, and its structural and physical characteristics have fascinated biophysicists. As an important membrane structural component in eukaryotic cells, cholesterol plays a critical role in maintenance of the semipermeable barrier between cell compartments, as well as in membrane fluidity. In addition to its important structural role, cholesterol modulates the functions of membrane proteins and the intracellular traffic of vesicles, participates in various transmembrane signaling processes (such as via G-protein-coupled receptors), and acts as precursor in the biosynthesis of vitamins and steroid hormones [9].

Mammalian cells can obtain cholesterol mainly via two routes. Cholesterol can be synthesized from acetyl-CoA through the mevalonate pathway [10], which also leads to the production of several isoprenoids, including farnesyl and geranylgeranyl lipids [11], taken up by low-density lipoprotein (LDL) endocytosis [12]. In addition, cholesterol is a key regulator of the mevalonate pathway in vertebrate cells [10]. Conversely, invertebrate cells do not synthesize cholesterol de novo [13], and the final product in the mevalonate pathway in insects constitutes juvenile hormones [14].

Cholesterol is distributed heterogeneously among cell membranes. In the plasma membrane, it represents 20–25% of the lipids. It is also abundant in endocytic compartments and the Golgi complex [15]. Conversely, the endoplasmic reticulum presents a low cholesterol content (less than 1% of total cellular cholesterol) [16]. In addition, cholesterol is important in the pathophysiology of different clinical conditions, such as in cardiac pathogenesis and cerebrovascular diseases, in the various forms of dementia, diabetes, and cancer, and in several rare monogenic diseases [15,17].

Recently, numerous studies addressed the role of cholesterol present in both the plasma or endosomal membrane and the viral particle envelope for the success of some viral infections. The infectivity of influenza virus [18] and human herpesvirus 6 (HHV-6) [19] is affected by depletion of cholesterol from their envelopes. Vaccinia virus, varicella-zoster virus, pseudorabies virus (PRV), and some arenaviruses were shown to be dependent on plasma membrane cholesterol for its efficient internalization into host cells [20,21,22,23]. Dependence on cholesterol from the cell membrane and/or the viral envelope has been demonstrated for different viruses, such as human immunodeficiency virus (HIV), herpes simplex virus (HSV), and hepatitis C virus (HCV) [24,25,26,27,28,29,30,31]. The role of cholesterol in different steps (attachment, fusion, and assembly) during flavivirus infection was addressed in a recent review [32]. Due to the importance of this lipid during viral infection, it is not surprising that some viruses modulate cholesterol metabolism to increase their chances of a more efficient infection.

In fact, during viral infection, cholesterol concentration in the host cell is modified, leading to alterations in the activity of different enzymes of the mevalonate pathway and favoring the formation of specific sites inside the host cell called “viral replication organelles” [33]. These organelles show high levels of cholesterol and other lipids, functioning as a viral replication-specific platform.

Cholesterol may be important in specific steps of viral infection and unnecessary in others. The presence of cholesterol in the envelope of some viruses, such as hepatitis B virus (HBV), is not required for virus binding to the host cell, but it is indispensable for the entry process since it can be an important factor in the viral fusion process [34]. In another example, cholesterol molecules in the viral envelope are necessary for efficient infection by canine distemper virus (CDV), while there are no changes in infectivity when it is removed from the host cell [35]. Despite these observations, the cholesterol present in either the cell membrane or the viral envelope contributes to replication by acting as a key component in the entry of enveloped viruses. However, cholesterol present in the cell membrane sometimes decreases the infectivity of some viruses, such as observed for rabies virus (RABV), where a reduction in cell cholesterol content may promote an increase in virus infectivity [36]. Overall, cholesterol may play an essential role at many stages of the virus infection process, mainly during the early events that lead to particle internalization (e.g., membrane fusion) and the formation of viral replication organelles [33,37,38].

## 3. Involvement of Cholesterol during Entry and Fusion of Alphaviruses

Alphaviruses enter into host cells through receptor-mediated endocytosis, followed by fusion of the viral envelope and the endosomal membrane [39]. When these vesicles mature in endosomes, the low pH inside them induces conformational changes in the envelope glycoproteins. These alterations include dissociation of the original heterodimer formed by E1/E2 glycoproteins and the consequent formation of homotrimers composed of E1. Alphaviruses are dependent on the presence of specific lipids in the host cell membrane so that the early events leading to nucleocapsid uncoating can occur. Among these lipids, cholesterol stands out. The first step of viral infection is binding to specific receptors or attachment molecules on the plasma membrane. Sometimes, these receptors/attachment molecules are localized in regions with a high cholesterol content (lipid rafts) [40,41]. Although alphaviruses may use different receptors to gain access into different host cells, these receptors do not seem to be localized in these regions. However, incoming particles of Mayaro virus (MAYV), a New World alphavirus, can colocalize with vesicles containing caveolin-1, an important raft marker, suggesting that alphaviruses can use domains with high cholesterol content to enter into the host cell [42]. In addition, there was a report where detergent-resistant microdomains (DRM), membranes derived from lipid rafts obtained from living cells, interacted efficiently with MAYV particles [43]. These findings suggest that alphaviruses may use cholesterol-rich domains for binding and internalization into the host cell.

The fusion properties and cholesterol dependence of alphaviruses have been studied in detail, mainly for the prototypical species Semliki forest virus (SFV) and SINV [44]. After internalization inside the endosome compartment, the E1 glycoprotein undergoes low-pH-dependent conformational changes that lead to the exposition of a fusion peptide and the interaction between the viral envelope and the endosomal membrane. Insertion of the fusion peptide of SFV and SINV is strongly favored by the presence of cholesterol in the target membrane [45,46,47]. In fact, in line with these findings, a previous study demonstrated that the E1 glycoprotein of SFV can directly bind to cholesterol, unlike that observed for flaviviruses, suggesting a possible role of cholesterol in the interaction between the viral envelope and the cellular membrane [48]. On the other hand, another study using sterols with different capacities to promote microdomain formation showed that SFV and SINV do not require cholesterol-rich domains for fusion with target membranes [49]. Membrane fusion and cholesterol requirement were recently demonstrated for another alphavirus, CHIKV, where it was found that the fusion rate changes according to cholesterol concentration [50,51]. Many of these studies on membrane fusion during the early events of alphavirus infection were based on liposomes or other artificial membrane systems composed of various lipids. Although they provide an important understanding of the cholesterol requirement for membrane fusion, these models exhibit some caveats, such as the absence of receptors and a high relative concentration of cholesterol.

Cholesterol dependence during alphavirus entry has also been demonstrated in cells using methyl-β-cyclodextrin (MβCD), a drug that promotes cholesterol depletion from membranes, or Cab-O-Sil, a colloidal silica used for cholesterol removal from serum [52], using invertebrate cells that are cholesterol auxotrophs [13]. Several studies demonstrated for SFV and SINV that cholesterol-depleted cells show a decrease of almost five logs in the levels of this lipid compared with control cells [52,53,54]. It is worth mentioning that cholesterol repletion reverses the inhibitory effect of its absence during viral infection [52]. Recently, the effect of cholesterol removal from the host cell on viral replication was revealed for other alphaviruses, such as MAYV and CHIKV [42,55], showing a cholesterol dependence during the early steps of infection. Interestingly, this cholesterol dependence is reduced in SFV mutants selected for growth in cholesterol-depleted cells (SRF—sterol requirement in function), although such a variant has not been identified naturally [54].

Conversely, a study with Venezuelan equine encephalitis virus (VEEV), a New World alphavirus, revealed a low sensibility to cholesterol depletion from mammalian cells during virus entry and viral RNA release from late endosomes [56]. Moreover, studies performed with CHIKV demonstrated a lack of correlation with cholesterol dependence in host cells during viral infection [57]. Taken together, these data suggest that cholesterol dependence cannot be a common characteristic for all alphaviruses and that host/viral factors should be considered (Table 1). However, this apparent conflict of cholesterol dependence could be explained not only by the different amino acids at position 226 that constitutes the ij loop of the E1 glycoprotein, but also by the amino-acid composition of the adjacent sequence [53,54]. Indeed, at the beginning of the recent CHIKV outbreak, E1-A226 viruses were isolated; however, as the epidemic progressed, E1-A226V mutants were preferentially isolated [58]. This mutation resulted in the virus becoming more dependent on the presence of cholesterol in the mosquito cell (C6/36) membrane, in addition to increasing the virus’s capability to replicate and disseminate into secondary organs of the mosquito vector [59]. However, it is still not clear whether there is a correlation between the cholesterol dependence and the increased fitness of CHIKV in C6/36 cells. Other studies are necessary to understand the effect of this mutation on viral fitness and contribute to the current knowledge regarding the role of cholesterol during alphavirus infection in different hosts.

## 4. Cholesterol Dependence during Post-Entry and Release Events of Alphaviruses

After binding to the receptor and entry of the viral particle into the host cell, the viral RNA is released and replicated. This process depends on intracellular membranes, and abnormalities in lipid composition/metabolism are likely to impact viral production at multiple steps. These modifications can be observed by manipulating key enzymes associated with the tricarboxylic acid cycle and mevalonate pathway, which modulate cholesterol synthesis. Indeed, numerous studies shed light on the relationship between lipid (specifically cholesterol) metabolism and arbovirus replication, mainly for members belonging to the *Flavivirus* genus [32,62].

Although cholesterol plays a critical role during different steps of alphavirus replication, studies on lipid metabolism and alphavirus replication are scarce. The importance of intracellular cholesterol during alphavirus infections was demonstrated through infection of Niemann–Pick disease A fibroblasts (NPAFs) with SINV [63]. NPAFs accumulate large amounts of cholesterol and sphingomyelin in the late endosomes and lysosomes localized in the perinuclear region. Virions produced in NPAFs are 26 times more infectious than viral particles budding from normal human fibroblasts (NHFs) [63]. However, virus infection in NPAFs results in reduced levels of genomic RNA and a lower ratio of viral subgenomic/genomic RNA, suggesting that the formation of replication complexes is not accomplished and indicating the importance of cholesterol during this process [63]. Likewise, a study performed with CHIKV using the drugs U18666A and imipramine, which led to cholesterol accumulation in endosome/lysosome compartments and an inhibition of cholesterol biosynthesis, revealed that the virus is unable to replicate under these conditions [64]. In parallel, during CHIKV infection, nsP1 partitions into cholesterol-rich DRMs, and its palmitoylated cysteines seem to be major players in this process [65]. It has also been observed that alphavirus infection leads to the activation of phosphatidylinositol 3-kinase (PI3K)/protein kinase B (AKT), resulting in an increase in glycolytic activity followed by a rise in acetyl-CoA concentration in infected cells [66]. The extensive and rapid increase in glycolysis observed upon alphavirus infection may result in an increase in cholesterol synthesis. Further studies must be conducted to ascertain this putative change in cholesterol metabolism.

Recent proteomic data also indicated perturbations in cholesterol content during CHIKV infection and suggested a downregulation at the level of gene expression of the enzymes associated with its metabolism/transport, such as hydroxymethylglutaryl-coenzyme A synthase (HMGCS1) and 3-hydroxy-3-methylglutaryl-coenzyme A reductase (HMGCR), in different host cells [67,68,69]. In addition, these data are in line with recent evidence showing an interferon-based antiviral mechanism, which impaired cholesterol biosynthesis in SFV-infected cells [69]. Taken together, these data suggested that alphavirus infection leads to a lower expression of key components associated with cholesterol biosynthesis, unlike that observed for other arboviruses, such as flaviviruses [32].

**Figure 1:**
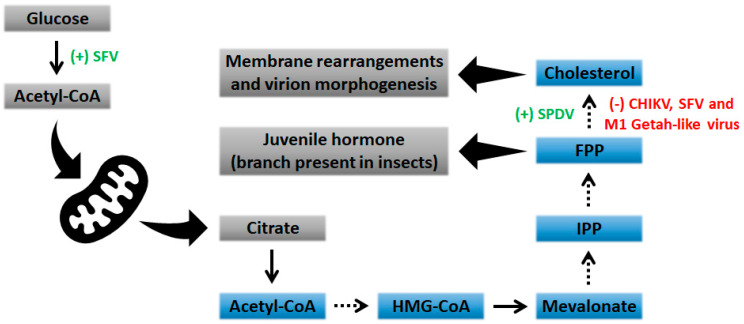
Effect of alphavirus infection on cholesterol biosynthesis. The mevalonate pathway (MVA) is a critical anabolic pathway to cholesterol biosynthesis. MVA pathway metabolites are shown in blue boxes. Dashed arrows represent multiple steps in the MVA pathway. The green symbol (+) denotes a stimulated pathway. The red symbol (-) denotes a blocked pathway. In order to simplify the scheme, many reactions and their reversibility are omitted. FPP, farnesyl diphosphate; HMG-CoA, 3-hydroxy-3-methylglutaryl coenzyme A; IPP, isopentenyl diphosphate; TCA, tricarboxylic acid cycle; SFV, Semliki forest virus; CHIKV, Chikungunya virus; SPDV, Salmon pancreas disease virus.

Interestingly, components of the cholesterol biosynthesis pathway seem to be downregulated not only by alphaviruses that are pathogenic to humans, but also by alphaviruses that have not been linked to human illness. The M1 strain of Getah-like alphavirus was isolated from *Culex* mosquitoes and is known to infect horses and pigs [70,71]. Recent studies demonstrated that M1 possesses selective and potent oncolytic activity, in addition to not being pathogenic, leaving normal cells intact [72,73]. Regarding cholesterol metabolism, more than 60% of genes associated with the cholesterol biosynthesis/mevalonate pathway are downregulated during M1 infection in different cell lines [74]. Conversely, Salmon pancreas disease virus (SPDV), an alphavirus that cause lesions in the pancreas, kidney, and cardiac and skeletal muscles of the infected fish, seems to modify the expression of genes involved in cholesterol metabolism [75,76]. Indeed, some gene transcripts involved at different steps of cholesterol biosynthesis were slightly upregulated during the early stages of SPDV infection [75]. It is worth noting that many of these experiments did not evaluate the activity of key enzymes related to cholesterol metabolism. Thus, the upregulation of transcripts, which leads to production of important enzymes in cholesterol biosynthesis, does not definitively indicate an increase in their activity and, therefore, further studies must be performed to elucidate the regulation of the mevalonate pathway at different steps during alphavirus infection. The different effects on cholesterol biosynthesis in the mevalonate pathway are shown in Figure 1.

Regarding alphavirus budding and release from the host cell, numerous studies reported that cholesterol plays a critical role in these events [54,60,61]. Studies performed with vertebrate and invertebrate cells demonstrated a similar requirement of cholesterol for viral release during infection by SFV and SIN (Table 1) [54,60,61]. Budding of viral particles is also restored by cholesterol repletion in infected cells. The inhibitory mechanism that blocks the efficient budding of viral particles seems to be due to a rapid and continued degradation of viral spike proteins in sterol-deficient cells [61]. However, more studies must be performed with alphaviruses that infect vertebrate hosts other than humans to comprehend the exact inhibitory mechanism associated with alphavirus budding.

## 5. Role Played by Cholesterol in the Alphavirus Particle

**Figure 2:**
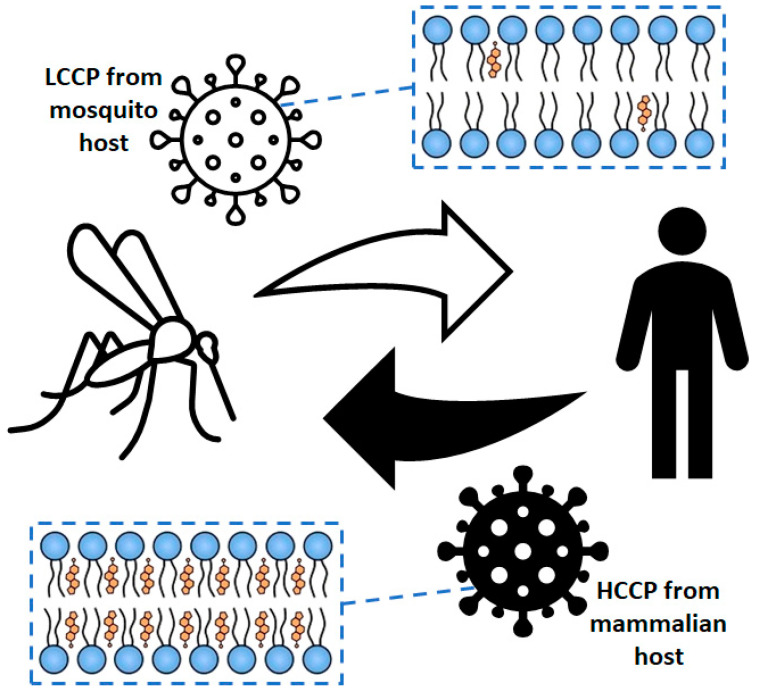
Envelope lipid packing from alphavirus particles isolated from mammalian and mosquito cells. LCCP, low-cholesterol content particles; HCCP, high-cholesterol content particles. Orange denotes cholesterol and blue denotes phospholipids.

In general, the lipid composition of the viral envelope reflects that observed in the host cell. Although some cells have a cholesterol/phospholipid molar ratio close to 1 [77], such a parameter in alphavirus particles isolated from vertebrate cells is sometimes higher than that found in plasma membranes [78,79]. Several studies demonstrated that cholesterol plays an important role in the stability, lateral organization, and packing of lipids in biological membranes [77,80,81]. Interestingly, the envelope of MAYV particles obtained from mosquito cells revealed a high level of lateral organization, as well as virus particles isolated from mammalian cells, despite the striking differences in cholesterol content (Figure 2) [82]. However, even though the envelope of viral particles isolated from vertebrate cells shows a high cholesterol level, this lipid is not critical for the biological activity of alphaviruses as it is for lateral organization of the viral envelope [82].

Concerning lipids other than cholesterol, alphaviruses such as SFV seem to have some selectivity for sphingomyelin species with long fatty-acid tails during budding from the host cell, since these lipids are up to fivefold more concentrated in the virus envelope than in the host cell plasma membrane [83]. A recent mass-spectrometry-based study revealed that SINV derived from mammalian cells has a substantially higher mass than SINV derived from insect cells because there is a higher portion of lipids containing long-chain fatty acids in the viral envelope [84]. As with the difference in cholesterol content, this difference in the extension of fatty-acid chains could also influence organization of the lipid bilayer and, ultimately, virus infectivity. Indeed, SINV progeny produced by mammalian cell lines (e.g., BHK-21) may contain less than 2% infectious particles, being more than an order of magnitude less infectious than SINV produced from mosquito cell lines (e.g., C6/36), as quantified by fluorescent foci-forming assays [84].

## 6. Concluding Remarks

Cholesterol is a key component of membranes in many different cell types. As viruses take advantage of membranes for their entry, replication, and exit from host cells, cholesterol becomes an important player in a range of infection processes for both enveloped and nonenveloped viruses. These roles may be even more important for enveloped viruses since their lipidic envelope is taken from the host cell when they bud from either plasmatic or endoplasmic membranes.

**Figure 3:**
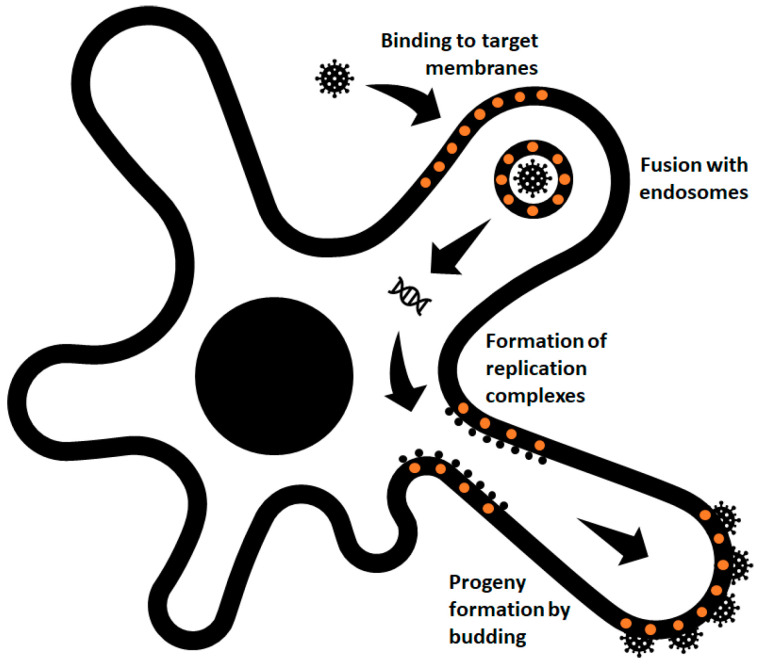
Overview of cholesterol functions in the life cycle of alphaviruses. Cholesterol molecules (orange circles) are required in different steps of both early (binding and fusion) and late (replication and budding) events of alphavirus infection in the host cell.

Alphaviruses are enveloped viruses that enter cells via receptor-mediated endocytosis and membrane fusion, releasing their RNA into the cell after endocytosis. During replication, membranous structures are assembled inside the cell, where virus protein synthesis and RNA replication take place. New progeny particles bud from the plasma membrane into the cell exterior carrying a small piece of the plasma membrane as their envelope. Accordingly, the membrane and its composition are quite important in the entire infection cycle of alphaviruses and, therefore, as is cholesterol. Cholesterol is not only important as a component determining the membrane physicochemical characteristics, but also as a molecular factor important for binding of viral surface proteins and their functions during virus entry [44] or as a structural factor contributing to the stability of virus particles [82,85]. Figure 3 summarizes these distinct roles played by cholesterol molecules in the life cycle of alphaviruses.

Nevertheless, the many different functional roles played by cholesterol in the stability, infectivity, and assembly of enveloped RNA viruses are not fully understood. Thus, due to its importance in virus–cell interactions, cholesterol can also be an interesting target in antiviral strategies [86], and pinpointing the influence of cholesterol on the success of virus infection has become an area of great interest in recent years. Interestingly, as arboviruses, the members of the *Alphavirus* genus are able to infect and replicate in mammalian and insect hosts, organisms that show very different cholesterol requirements in their physiology and, consequently, very different cholesterol concentrations in their cells. Irrespective of the host cell and despite the quite different cholesterol content, MAYV progeny particles were shown to be equally infectious and stable [82].

While the mild effects of alphavirus infection in invertebrate hosts suggest an ancient evolutionary relationship between these viruses and the sterol-poor cells of these hosts, the successful infection of vertebrate hosts and the production of infectious particles capable of recycling between mammals and insects suggest that metabolic and molecular factors in the latter’s cells should be able to overcome the cholesterol requirement of these viruses presented in the current scientific literature. Once again, this indicates that this is an area yet to be explored, which may lead to an understanding not only of the role of cholesterol, but also of the lipid–lipid and lipid–protein interactions in viral infection processes.

## Figures and Tables

**Table 1:** Cholesterol requirement during different steps of the life cycle of specific alphaviruses.

Alphavirus *	Binding	Fusion	Replication	Budding	References
CHIKV	ND	+	+	ND	[50,51]
MAYV	+	+	ND	ND	[42,43]
SFV	+	+	ND	+	[44,45,46,48,54,60,61]
SINV	+	+	ND	+	[44,47,48]
VEEV	-	-	ND	ND	[56]

* No data available for other alphaviruses. CHIKV, Chikungunya virus; MAYV, Mayaro virus; SFV, Semliki forest virus; SINV, Sindbis virus; VEEV, Venezuelan equine encephalitis virus; +, required; -, not required; ND, not determined.

## References

[1] Paredes A., Weaver S., Watowich S., Chiu W. (2005). Structural biology of old world and new world alphaviruses. Arch. Virol..

[2] Strauss J.H., Strauss E.G. (1994). The alphaviruses: Gene expression, replication, and evolution. Microbiol. Rev..

[3] Chen R., Mukhopadhyay S., Merits A., Bolling B., Nasar F., Coffey L.L., Powers A., Weaver S.C. (2018). ICTV Virus Taxonomy Profile: Togaviridae. J. Gen. Virol..

[4] Paredes A.M., Simon M.N., Brown D.T. (1992). The mass of the Sindbis virus nucleocapsid suggests it has T = 4 icosahedral symmetry. Virology.

[5] Ramsey J., Mukhopadhyay S. (2017). Disentangling the Frames, the State of Research on the Alphavirus 6K and TF Proteins. Viruses.

[6] Kääriäinen L., Ahola T. (2002). Functions of alphavirus nonstructural proteins in RNA replication. Prog. Nucleic Acid Res. Mol. Biol..

[7] Schuchman R., Kilianski A., Piper A., Vancini R., Ribeiro J., Sprague T.R., Nasar F., Boyd G., Hernandez R., Glaros T. (2018). Comparative Characterization of the Sindbis Virus Proteome from Mammalian and Invertebrate Hosts Identifies nsP2 as a Component of the Virion and Sorting Nexin 5 as a Significant Host Factor for Alphavirus Replication. J. Virol..

[8] Hafer A., Whittlesey R., Brown D.T., Hernandez R. (2009). Differential incorporation of cholesterol by Sindbis virus grown in mammalian or insect cells. J. Virol..

[9] Cherezov V., Rosenbaum D.M., Hanson M.A., Rasmussen S.G., Thian F.S., Kobilka T.S., Choi H.J., Kuhn P., Weis W.I., Kobilka B.K. (2007). High-resolution crystal structure of an engineered human β2-adrenergic G protein-coupled receptor. Science.

[10] Goldstein J.L., Brown M.S. (1990). Regulation of the mevalonate pathway. Nature.

[11] Zhang F.L., Casey P.J. (1996). Protein prenylation: Molecular mechanisms and functional consequences. Annu. Rev. Biochem..

[12] Goldstein J.L., Brown M.S., Anderson R.G.W., Russell D.W., Schneider W.J. (1985). Receptor-mediated endocytosis: Concepts emerging from the LDL receptor system. Annu. Rev. Cell. Biol..

[13] Cark A.J., Block K. (1959). The absence of sterol synthesis in insects. J. Biol. Chem..

[14] Bellés X., Martín D., Piulachs M.D. (2005). The mevalonate pathway and synthesis of juvenile hormone in insects. Annu. Rev. Entomol..

[15] Maxfield F.R., Tabas I. (2005). Role of cholesterol and lipid organization in disease. Nature.

[16] Ikonen E. (2008). Cellular cholesterol trafficking and compartmentalization. Nat. Mol. Cell Biol..

[17] Ikonen E. (2006). Mechanisms for cellular cholesterol transport: Defects and human disease. Physiol. Rev..

[18] Sun X., Whittaker G.R. (2003). Role for influenza virus envelope cholesterol in virus entry and infection. J. Virol..

[19] Huang H., Li Y., Sadaoka T., Tang H., Yamamoto T., Yamanishi K., Mori Y. (2006). Human herpesvirus 6 envelope cholesterol is required for virus entry. J. Gen. Virol..

[20] Chung C.S., Huang C.Y., Chang W. (2005). Vaccinia virus penetration requires cholesterol and results in specific viral envelope proteins associated with lipid rafts. J. Virol..

[21] Hambleton S., Steinberg S.P., Gershon M.D., Gershon A.A. (2007). Cholesterol dependence of varicella-zoster virion entry into target cells. J. Virol..

[22] Desplanques A.S., Nauwynck H.J., Vercauteren D., Geens T., Favoreel H.W. (2008). Plasma membrane cholesterol is required for efficient pseudorabies virus entry. Virology.

[23] Vela E.M., Zhang L., Colpitts T.M., Davey R.A., Aronson J.F. (2007). Arenavirus Entry occurs through a cholesterol-dependent, non-caveolar, clathrin-mediated endocytic mechanism. Virology.

[24] Bender F.C., Whitbeck J.C., Ponce De Leon M., Lou H., Eisenberg R.J., Cohen G.H. (2003). Specific association of glycoprotein B with lipid rafts during herpes simplex virus entry. J. Virol..

[25] Campbell S.M., Crowe S.M., Mak J. (2001). Lipid rafts and HIV-1: From viral entry to assembly of progeny virions. J. Clin. Virol..

[26] Campbell S.M., Crowe S.M., Mak J. (2002). Virion-associated cholesterol is critical for the maintenance of HIV-1 structure and infectivity. AIDS.

[27] Graham D.R., Chertova E., Hilburn J.M., Arthur L.O., Hildreth J.E. (2003). Cholesterol depletion of human immunodeficiency virus type 1 and simian immunodeficiency virus with β-cyclodextrin inactivates and permeabilizes the virions: Evidence for virion-associated lipid rafts. J. Virol..

[28] Guyader M., Kiyokawa E., Abrami L., Turelli P., Trono D. (2002). Role for human immunodeficiency virus type 1 membrane cholesterol in viral internalization. J. Virol..

[29] Viard M., Parolini I., Sargiacomo M., Fecchi K., Ramoni C., Ablan S., Ruscetti F.W., Wang J.M., Blumenthal R. (2002). Role of cholesterol in human immunodeficiency virus type 1 envelope protein mediated fusion with host cells. J. Virol..

[30] Aizaki H., Morikawa K., Fukasawa M., Hara H., Inoue Y., Tani H., Saito K., Nishijima M., Hanada K., Matsuura Y. (2008). Critical role of virion-associated cholesterol and sphingolipid in hepatitis C virus infection. J. Virol..

[31] Jeon J.H., Lee C. (2018). Cholesterol is important for the entry process of porcine deltacoronavirus. Arch. Virol..

[32] Osuna-Ramos J.F., Reyes-Ruiz J.M., Del Ángel R.M. (2018). The role of host cholesterol during flavivirus infection. Front. Cell. Infect. Microbiol..

[33] Zhang Z., He G., Filipowicz N.A., Randall G., Belov G.A., Benjamin G., Wang X. (2019). Host lipids in positive-strand RNA virus genome replication. Front. Microbiol..

[34] Bremer C.M., Bung C., Kott N., Hardt M., Glebe D. (2008). Hepatitis B virus infection is dependent on cholesterol in the viral envelope. Cell. Microbiol..

[35] Imhoff H., Von Messling V., Herrler G., Haas L. (2007). Canine distemper virus infection requires cholesterol in the viral envelope. Virology.

[36] Hotta K., Bazartseren B., Kaku Y., Noguchi A., Okutami A., Inoue S., Yamada A. (2008). Effect of cellular cholesterol depletion on rabies virus infection. Virus Res..

[37] Teissier E., Pécheur E.I. (2007). Lipids as modulators of membrane fusion mediated by viral fusion proteins. Eur. Biophys. J..

[38] Strating J.R., van Kuppeveld F.J. (2017). Viral rewiring of cellular lipid metabolism to create membranous replication compartments. Curr. Opin. Cell Biol..

[39] Helenius A., Kartenbeck J., Simons K., Fries E. (1980). On the entry of Semliki forest virus into BHK-21 cells. J. Cell Biol..

[40] Chazal N., Gerlier D. (2003). Virus entry, assembly, budding, and membrane rafts. Microbiol. Mol. Biol. Rev..

[41] Takahashi T., Suzuki T. (2011). Function of membrane rafts in viral life cycles and host cellular response. Biochem. Res. Int..

[42] Carvalho C.A.M., Silva J.L., Oliveira A.C., Gomes A.M.O. (2017). On the entry of an emerging arbovirus into host cells: Mayaro virus takes the highway to the cytoplasm through fusion with early endosomes and caveolae-derived vesicles. PeerJ.

[43] Sousa I.P., Carvalho C.A.M., Mendes Y.S., Weissmuller G., Oliveira A.C., Gomes A.M.O. (2017). Fusion of a new world alphavirus with membrane microdomains involving partially reversible conformational changes in the viral spike proteins. Biochemistry.

[44] Kielian M., Chanel-Vos C., Liao M. (2010). Alphavirus entry and membrane fusion. Viruses.

[45] Ahn A., Gibbons D., Kielian M. (2002). The fusion peptide of Semliki Forest Virus associates with sterol-rich membrane domains. J. Virol..

[46] Kielian M.C., Helenius A. (1984). Role of cholesterol in fusion of Semliki Forest virus with membranes. J. Virol..

[47] Smit J.M., Bittman R., Wilschut J. (1999). Low-pH-dependent fusion of Sindbis virus with receptor-free cholesterol-and-sphingolipid-containing liposomes. J. Virol..

[48] Umashankar M., Sánchez-San Martín C., Liao M., Reilly B., Guo A., Taylor G., Kielian M. (2008). Differential cholesterol binding by class ii fusion proteins determines membrane fusion properties. J. Virol..

[49] Waarts B.L., Bittman R., Wilschut J. (2002). Sphingolipid and cholesterol dependence of alphavirus membrane fusion. Lack of correlation with lipid raft formation in target liposomes. J. Biol. Chem..

[50] Van Duijl-Richter M.K., Hoornweg T.E., Rodenhuis-Zybert I.A., Smit J.M. (2015). Early events in chikungunya virus infection—From virus cell binding to membrane fusion. Viruses.

[51] Hoornweg T.E., van Duijl-Richter M.K.S., Ayala Nuñez N.V., Albulescu I.C., van Hemert M.J., Smit J.M. (2016). Dynamics of Chikungunya Virus Cell Entry Unraveled by Single-Virus Tracking in Living Cells. J. Virol..

[52] Phalen T., Kielian M. (1991). Cholesterol is required for infection by Semliki Forest virus. J. Cell Biol..

[53] Lu Y.E., Cassese T., Kielian M. (1999). The cholesterol requirement for Sindbis virus entry and exit and characterization of a spike protein region involved in cholesterol dependence. J. Virol..

[54] Vashishtha M., Phalen T., Marquardt M.T., Ryu J.S., Ng A.C., Kielian M. (1998). A single point mutation controls the cholesterol dependence of Semliki Forest virus entry and exit. J. Cell Biol..

[55] Bernard E., Solignat M., Gay B., Chazal N., Higgs S., Devauux C., Briant L. (2010). Endocytosis of chikungunya virus into mammalian cells: Role of clathrin and early endosomal compartments. PLoS ONE.

[56] Kolokoltsov A.A., Fleming E.H., Davey R.A. (2006). Venezuelan equine encephalitis virus entry mechanism requires late endosome formation and resists cell membrane cholesterol depletion. Virology.

[57] Tsetsarkin K.A., McGee C.E., Higgs S. (2011). Chikungunya virus adaptation to Aedes albopictus mosquitoes does not correlate with acquisition of cholesterol dependence or decreased pH threshold for fusion reaction. Virol. J..

[58] Schuffenecker I., Iteman I., Michault A., Murri S., Frangeul L., Vaney M.C., Lavenir R., Pardigon N., Reynes J.M., Pettinelli F. (2006). Genome microevolution of chikungunya viruses causing the Indian Ocean outbreak. PLoS Med..

[59] Tsetsarkin K.A., Vanlandingham D.L., McGee C.E., Higgs S. (2007). A single mutation in chikungunya virus affects vector specificity and epidemic potential. PLoS Pathog..

[60] Marquardt M.T., Phalen T., Kielian M. (1993). Cholesterol is required in the exit pathway of Semliki Forest virus. J. Cell Biol..

[61] Lu Y.E., Kielian M. (2000). Semliki forest virus budding: Assay, mechanisms, and cholesterol requirement. J. Virol..

[62] Mayer K.A., Stöckl J., Zlabinger G.J., Gualdoni G.A. (2019). Hijacking the supplies: Metabolism as a novel facet of virus-host interaction. Front. Immunol..

[63] Ng C.G., Coppens I., Govindarajan D., Pisciotta J., Shulaev V., Griffin D.E. (2008). Effect of host cell lipid metabolism on alphavirus replication, virion morphogenesis, and infectivity. Proc. Natl. Acad. Sci. USA.

[64] Wichit S., Hamel R., Bernard E., Talignani L., Diop F., Ferraris P., Liegeois F., Ekchariyawat P., Luplertlop N., Surasombatpattana P. (2017). Imipramine inhibits chikungunya virus replication in human skin fibroblasts through interference with intracellular cholesterol trafficking. Sci. Rep..

[65] Bakhache W., Neyret A., Bernard E., Merits A., Briant L. (2020). Palmitoylated cysteines in Chikungunya virus nsP1 are critical for targeting to cholesterol-rich plasma membrane microdomains with functional consequences for viral genome replication. J. Virol..

[66] Mazzon M., Castro C., Thaa B., Liu L., Mutso M., Liu X., Mahalingam S., Griffin J.L., Marsh M., McInerney G.M. (2018). Alphavirus-induced hyperactivation of PI3K/AKT directs pro-viral metabolic changes. PLoS Pathog..

[67] Thio C.L., Yusof R., Abdul-Rahman P.S., Karsani S.A. (2013). Differential proteome analysis of chikungunya virus infection on host cells. PLoS ONE.

[68] Abere B., Wikan N., Ubol S., Auewarakul P., Paemanee A., Kittisenachai S., Roytrakul S., Smith D.R. (2012). Proteomic Analysis of Chikungunya Virus Infected Microgial Cells. PLoS ONE.

[69] Blanc M., Hsieh W.Y., Robertson K.A., Watterson S., Shui G., Lacaze P., Khondoker M., Dickinson P., Sing G., Rodríguez-Martín S. (2011). Host defense against viral infection involves interferon mediated down regulation of sterol biosynthesis. PLoS Biol..

[70] Wen J.S., Zhao W.Z., Liu J.W., Zhou H., Tao J.P., Yan H.J., Liang Y., Zhou J.J., Jiang L.F. (2007). Genomic analysis of a Chinese isolate of Getah-like virus and its phylogenetic relationship with other Alphaviruses. Virus Genes.

[71] Zhai Y.G., Wang H.Y., Sun X.H., Fu S.H., Wang H.Q., Attoui H., Tang Q., Liang G.D. (2008). Complete sequence characterization of isolates of Getah virus (genus Alphavirus, family Togaviridae) from China. J. Gen. Virol..

[72] Lin Y., Zhang H., Liang J., Li K., Zhu W., Fu L., Wang F., Zheng X., Shi H., Wu S. (2014). Identification and characterization of alphavirus M1 as a selective oncolytic virus targeting ZAP-defective human cancers. Proc. Natl. Acad. Sci. USA.

[73] Zhang H., Lin Y., Li K., Liang J., Xiao X., Cai J., Tan Y., Xing F., Mai J., Li Y. (2016). Naturally existing oncolytic virus M1 is nonpathogenic for the nonhuman primates after multiple rounds of repeated intravenous injections. Hum. Gene Ther..

[74] Liang J., Guo L., Li K., Xiao X., Zhu W., Zheng X., Hu J., Zhang H., Cai J., Yu Y. (2018). Inhibition of the mevalonate pathway enhances cancer cell oncolysis mediated by M1 virus. Nat. Commun..

[75] Herath T.K., Bron J.E., Thompson K.D., Taggart J.B., Adams A., Ireland J.H., Richards R.H. (2012). Transcriptomic analysis of the host response to early stage salmonid alphavirus (SAV-1) infection in Atlantic salmon Salmo salar L.. Fish Shellfish Immunol..

[76] Deperasińska I., Schulz P., Siwicki A.K. (2018). Salmonid Alphavirus (SAV). J. Vet. Res..

[77] Van Meer G., Voelker D.R., Feigenson G.W. (2008). Membrane lipids: Where they are and how they behave. Nat. Mol. Cell Biol..

[78] Laine R., Söderlund H., Renkonen O. (1973). Chemical composition of Semliki Forest virus. Intervirology.

[79] Renkonen O., Kääriäinen L., Simons K., Gahmberg C.G. (1971). The lipid class composition of Semliki Forest Virus and of plasma membranes of the host cells. Virology.

[80] Bagatolli L.A. (2006). To see or not to see: Lateral organization of biological membranes and fluorescence microscopy. Biochim. Biophys. Acta.

[81] Sonnino S., Prinetti A. (2010). Lipids and membrane lateral organization. Front. Physiol..

[82] Sousa I.P., Carvalho C.A., Ferreira D.F., Weissmüller G., Rocha G.M., Silva J.L., Gomes A.M. (2011). Envelope lipid-packing as a critical factor for the biological activity and stability of alphavirus particles isolated from mammalian and mosquito cells. J. Biol. Chem..

[83] Kalvodova L., Sampaio J.L., Cordo S., Ejsing C.S., Shevchenko A., Simons K. (2009). The lipidomes of vesicular stomatitis virus, semliki forest virus, and the host plasma membrane analyzed by quantitative shotgun mass spectrometry. J. Virol..

[84] Dunbar C.A., Rayaprolu V., Wang J.C., Brown C.J., Leishman E., Jones-Burrage S., Trinidad J.C., Bradshaw H.B., Clemmer D.E., Mukhopadhyay S. (2019). Dissecting the components of Sindbis virus from arthropod and vertebrate hosts: Implications for infectivity differences. ACS Infect. Dis..

[85] Bajimaya S., Frankl T., Hayashi T., Takimoto T. (2017). Cholesterol is required for stability and infectivity of influenza A and respiratory syncytial viruses. Virology.

[86] Martín-Acebes M.A., Jiménez de Oya N., Saiz J.C. (2019). Lipid metabolism as a source of druggable targets for antiviral discovery against Zika and other flaviviruses. Pharmaceuticals.

